# Telocytes and Their Structural Relationships With the Sperm Storage Tube and Surrounding Cell Types in the Utero-Vaginal Junction of the Chicken

**DOI:** 10.3389/fvets.2022.852407

**Published:** 2022-03-24

**Authors:** Xudong Zhu, Qi Wang, Piotr Pawlicki, Ziyu Wang, Bernadetta Pawlicka, Xiangfei Meng, Yongchao Feng, Ping Yang

**Affiliations:** ^1^College of Sciences, Nanjing Agricultural University, Nanjing, China; ^2^MOE Joint International Research Laboratory of Animal Health and Food Safety, College of Veterinary Medicine, Nanjing Agricultural University, Nanjing, China; ^3^Center of Experimental and Innovative Medicine, University of Agriculture in Krakow, Krakow, Poland; ^4^College of Animal Science and Technology, Nanjing Agricultural University, Nanjing, China; ^5^Laboratory of Genetics and Evolutionism, Institute of Zoology and Biomedical Research, Jagiellonian University, Krakow, Poland

**Keywords:** telocytes (TCs), chicken, utero-vaginal junction, sperm storage tube, ultrastructure

## Abstract

Telocytes (TCs) are a new type of mesenchymal cells that have been discovered recently in many organs and tissues. However, studies of TCs in the avian reproductive system are still at the beginning. Chickens are one of the world's most popular domesticated animals, providing inexpensive but valuable proteins and nutrients from chickens and eggs to nourish the human bodies. Chickens have important scientific value; thus, understanding the reproductive system regulations seems to be important. The utero-vaginal junction is involved in the regulation of sperm storage. The sperm storage tube (SST) in the utero-vaginal junction stores sperm. The purpose of this study was to investigate the existence of TCs in the utero-vaginal junction of the chicken, and their structural relationships with the sperm storage tube and surrounding cell types. We studied the morphology, ultrastructure, and immune characterization of TCs.

**Methods:**

The utero-vaginal junction of 4-month-old healthy adult chickens (*n* = 10) were used for Masson's staining, fluorescent *in situ* hybridization technique (FISH), and transmission electron microscopy (TEM) analysis. The results showed that TCs were present in the utero-vaginal junction. TCs appear as CD34 immunopositive and C-kit immunopositive. They were identified especially *via* small-body and long-protrusion telopodes (Tps) containing Podomers (Pm) and Podoms (Pd). The Tps were bent, folded, and intertwined with each other, sometimes in the shape of a labyrinth. The Tps were embedded between collagen fiber bundles, smooth muscle bundles, and around blood vessels and releasing vesicles. TCs surround these glands, forming heteromorphic cell connections with surrounding lymphocytes and plasma cells, smooth muscle cells, blood vessels, collagen fibers, and fibroblast-formed homotypic or allotypic connections in a complex three-dimensional network structure. This study provides a morphological basis for the possible role of TCs in regulating the utero-vaginal junction physiological role and in intercellular communication.

## Introduction

Telocytes (TCs) are a new type of mesenchymal cells named by Romanian Professor Popescu and Italian Professor Faussone in 2010. This cell type was first discovered in the human pancreas by Popescu and his team in 2005 because its form is similar to the interstitial cells of Cajal (ICCs) in the digestive tract. He speculated that these cells may be a form of ICC, called simple Cajal interstitial cells (interstitial Cajal like cells; ICLCs) (1). In 2008, Pieri et al. reported that ICLCs are completely different from ICCs in morphological structure, immunophenotype, and distribution characteristics by using electron microscopy, immunofluorescence, and immunohistochemistry techniques. Popescu keenly recognized that ICLCs are a new type of mesenchymal cell that had not been discovered. In 2010, Popescu and Faussone reported that TCs have extremely long protrusions, which are important markers to distinguish TCs from ICCs, fibroblasts, and dendritic cells. Because of their special shape, ICLCs were initially renamed to TCs in 2010 (2). Telocytes have a small oval body containing a nucleus surrounded by a small amount of cytoplasm. The nucleus is large and contains clusters of heterochromatins distributed over the nuclear membrane. There are 2–6 extremely long and thin (ranging from tens to hundreds of microns in length with internal diameters <0.2 μm) protrusions showing alternation of expanded segments (Pd) and elongated segments (Pm) in the shape of rosary beads. The enlarged segment contains vesicles, mitochondria, and rough endoplasmic reticulum, while the elongated segment rarely contains organelles (3).

Researchers have studied TCs in various organs of mammals, such as the heart, oviduct, small intestine, skin, myometrium, testis, and esophagus (4–8), but only a few studies have been performed on the chicken oviduct. TCs can be labeled by multiple antibodies, such as CD34, vimentin, c-kit, caveolin-1, and platelet-derived growth factor (PDGFR) receptor (9–17), but their immunophenotypes varied by species and organ. In the heart, TCs express CD34, CD28, vimentin, PDGFR-α, Sca-1, and c-kit but are negative for the hematopoietic marker CD45 (7, 13). As CD28 and vimentin are also expressed by cardiac mesenchymal stem cells, it is speculated that TCs may originate from differentiated cardiac mesenchymal stem cells. In the lungs, TCs are positive for c-kit, CD34, and vascular endothelial growth factor, suggesting that they may be involved in angiogenesis (18). In the female reproductive system, TCs express CD34, c-kit, PDGFR-α, T-type/the Ca^2+^ channel, and estrogen and progesterone receptors (7, 19). However, until today no single marker specific for TCs has been found; the immunophenotype of TCs is heterogeneous and it is generally believed that reliable immune markers for TCs include CD34 and PDGFR-α, double CD34, and PDGFR-α, or a double marker based on CD34 and PDGFR-α (2). The functions of TCs are less understood. TCs extend extremely long protrusions to form multiple junction points through homotypic and heterotypic junctions and a complex three-dimensional (3D) network structure in stromal tissue. Homotypic junctions are contacts between different TC processes, and heterotypic junctions are contacts between TCs and blood vessels and adjacent cells (such as cardiomyocytes, stem cells, fibroblasts, and immune cells) (2, 20). The structure of a cell determines its function, and the unique shape of TCs is linked with a variety of functional roles. TCs play roles in cell signaling, mechanical support, immune surveillance, tissue repair, and regeneration, and are potentially involved in a variety of diseases and physiological processes (21).

However, research on TCs in the female reproductive system of birds is still at the beginning. The utero-vaginal junction is involved in the regulation of sperm storage. The sperm storage tube in the utero-vaginal junction stores sperm. The fowl and mammalian oviducts are similar in that they are divided into a mucous membrane, a muscular layer, and a serosal layer (outer membrane). In contrast, the fowl oviduct is a unilateral organ, coiling in the abdominal cavity on the left side, and composed of the infundibulum, magnum, isthmus, uterine, and vagina. The Japanese scholar Fujii reported in 1963 that the oviduct and uterus and the integration of the vaginal mucosa lamina propria gland can store sperm (22). The oviduct at the front end of the vagina is referred to as the vagina gland and is a fine tubular sperm gland or sperm storage tube (23). The sperm storage tube is a tubular gland surrounded by a single row of columnar epithelial cells and is distributed in the lamina propria of the mucosa. The tubular gland opens on the surface of the longitudinal mucosal folds at the utero-vaginal junction. The opening is surrounded by pseudostratified columnar ciliated epithelium, and the gland is surrounded by loose connective tissue. This study explores the distribution and ultrastructure of TCs in the utero-vaginal junction, as well as the tissue structure and microenvironment of the junction with the participation of TCs and the particular spatial relationships with neighboring cells. This study will fill in the TC gaps in chicken oviduct research and provide a morphological basis for the possible role of TCs in regulating the utero-vaginal junction physiological role and in intercellular communication.

## Materials and Methods

### Animals

#### Sample

Ten healthy adult laying chickens, 4 months of age, weighing 2.0–2.5 kg per chicken were used in this study. The feed fed was commercial adult broiler feed (Chengdu New Hope Group, China). Healthy adult chickens were kept in a temperature-controlled room with natural light (20 ± 1°C) (light/dark period, 12/12 h), with free access to food and water. The chickens were kept and observed for 7 days. For sample collection, the birds were euthanized by cervical dislocation after intravenous administration of 3% sodium pentobarbital. The left oviduct was extracted immediately. The utero-vaginal junction was taken and fixed in 2.5% glutaraldehyde for transmission electron microscopy method. The utero-vaginal junction was fixed in 4% paraformaldehyde/PBS overnight for Masson staining. The utero-vaginal junction was fixed in the 4% paraformaldehyde/DEPC (G1113, Servicebio, Wuhan, China) above 12 h for fluorescent *in situ* hybridization. Tissue samples were dehydrated in a series of graded concentration ethanol (75, 85, 95, 95, 100, and 100%) (100092683, Sinopharm Chemical Reagent Co., Ltd., Shanghai, China). Tissue samples were first embedded and made into paraffin wax blocks, and then tissue sections were cut into 5 μm. Sample preparation was conducted according to accepted international standards.

### Masson Staining

Tissue sections were dewaxed, stained with Weigert ferroxylin staining solution, and differentiated with acidic ethanol differentiation solution. After sections were washed with water, the Masson blue solution returned to blue, followed by the lichunsin fuchsin staining solution, and then washed with molybdenum phosphomolybdic acid solution and the prepared weak acid working solution. Sections were dyed in aniline blue solution and washed in weak acid working solution. Dehydration was performed in ethanol and xylene (100092683, Sinopharm Chemical Reagent Co., Ltd.) and then cover-slipped (Masson's Trichrome Stain Kit; G1340; Solarbio, Beijing, China). The stained sections were analyzed using a light microscope (BX53; Olympus, Tokyo, Japan) equipped with a camera (DP73; Olympus).

### Fluorescent *in situ* Hybridization Technique

For FISH, firstly, the gene sequence of chicken CD34 and C-kit was obtained from GenBank, and the GenBank login number was GI: 2024484067(CD34), 303532(C-kit); the DNA STAR software was used to compare it with CD34 and C-kit gene sequences of other species to find the homologous sequence. BLAST analysis was performed on the NCBI website based on chicken the genome database to confirm the specificity of the homologous sequence, and then a specific reverse-phase three-oligonucleotide probe was designed. Fluorescence probe CD34 and C-Kit were synthesized by Wuhan Servicebio Technology Co., Ltd. The tissue sections were dewaxed, washed in DEPC dilution, followed by retrieval of the antigen epitope, and naturally cooled. Add proteinase K (20 μg/ml) (G1205, Servicebio, Wuhan, China) working solution to cover objectives and incubate at 37°C for 30 min. Wash in pure water, then wash three times in PBS (pH 7.4) (G0002, Servicebio, Wuhan, China) on a Rocker device, 5 min each. Add pre-hybridization solution to each section and incubate for 1 h at 37°C. Remove the pre-hybridization solution, add the fluorescence probe CD34 and C-kit hybridization solution with a concentration of 0.25 μg/ml, and incubate the section in a humidity chamber and hybridize overnight at 42°C. Remove the hybridization solution. Wash sections in 2 × SSC (G3016-4, Servicebio, Wuhan, China) for 10 min at 37°C. Wash sections in 1 × SSC two times for 5 min each at 37°C, and wash in 0.5 × SSC for 10 min at room temperature. Fluorescent staining was protected from light and sections were mounted with a mounting medium with 4′,6-diamidino-2-phenylindole (DAPI) (catalog no. AR1176; Boster Biotechnology, Wuhan, China). The sections were examined using a luminescence microscope (BX53; Olympus, Tokyo, Japan) equipped with a camera (DP73; Olympus).

### Transmission Electron Microscopy

Small sections of fresh tissue (1 mm^3^) were fixed in 2.5% glutaraldehyde PBS solution pre-cooled overnight at 4°C. Tissue fragments were then rinsed with 0.01 M PBS solution (pH = 7.4) and then post-fixed with 1% osmium tetroxide at room temperature for 1 h (Polysciences Inc., Warrington, PA, USA). Dehydration was done in ethanol; the material was embedded in Epon812 epoxy resin and polymerized at 60°C for 3 days. After the careful orientation of the 1-μm semi-thin section, the ultrathin sections (50 nm) were prepared and attached to the copper net. The ultrathin sections were stained with 1% uranyl acetate for 10 min and Reynold's lead citrate for 5 min. Ultrathin sections were analyzed with a Hitachi H-7650 transmission electron microscope (Japan).

## Results

### General Histology of the Utero-Vaginal Junction of the Chicken

**Figure 1:**
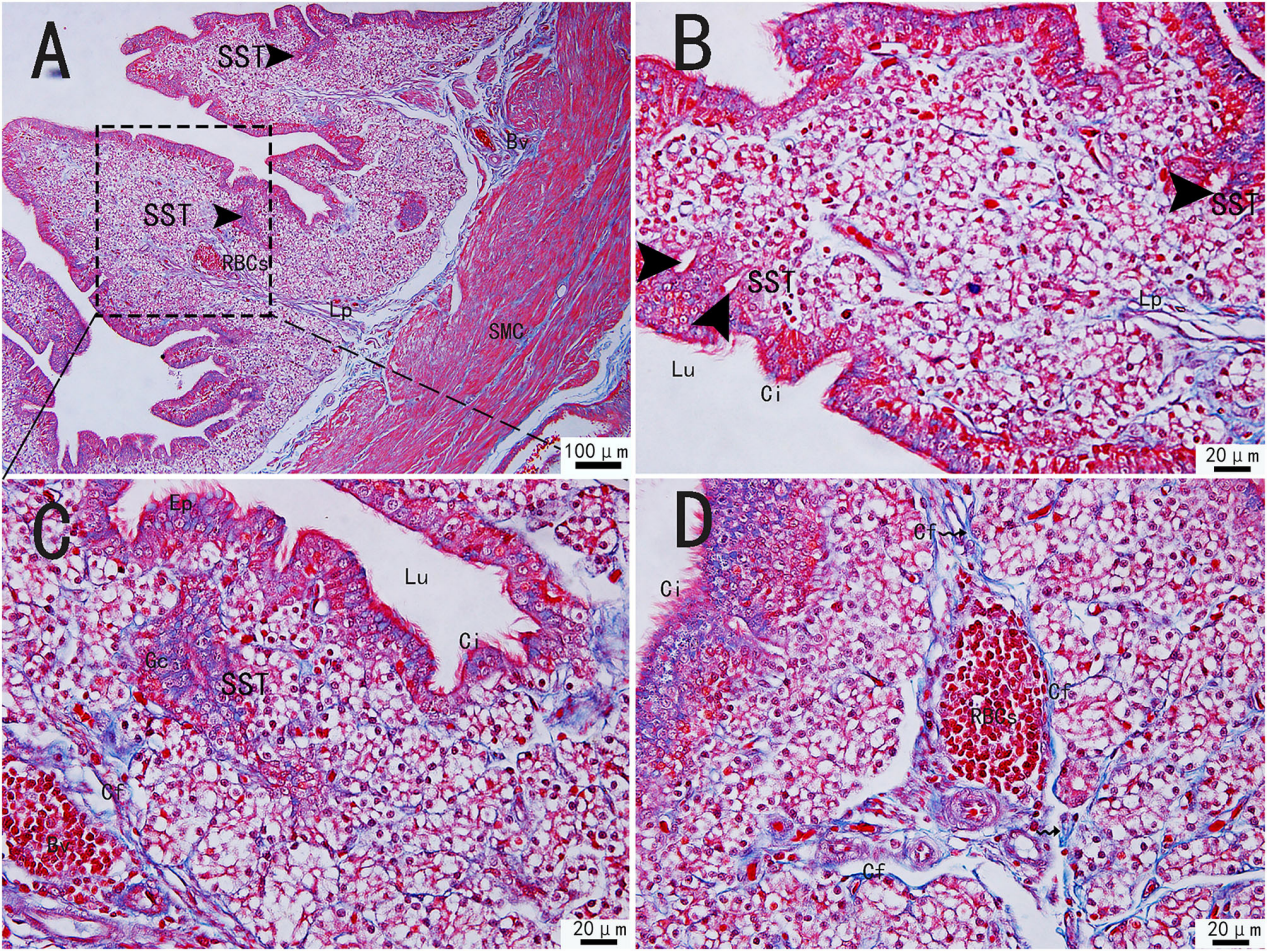
Morphological structure of the uterine-vaginal junction of the oviduct in chicken by Masson's staining. **(C)** The enlargement of the area marked by **(A)** dotted box. The sperm storage tube existed between the collagen fibers **(B)**. The blood vessels contained a large number of red blood cells and were surrounded by a large number of collagen fibers **(D)**. SMC, Smooth muscle; Lp, lamina propria; Ep, Epithelial cells; SST, Sperm storage tube (black triangular arrow); Gc, Gland cell; Bv, blood vessel; Ci, cilia; RBCs, red blood cells; Cf, Collagen fiber; Lu, lumen. Scale bar **(A)** = 100 μm; Scale bar **(B–D)** = 20 μm.

The collagen fibers were stained in blue, and the muscle fibers were stained in red by Masson's staining. Masson's staining revealed the mucosal, muscular, and serosal layers from inside to outside the utero-vaginal junction. The free surface of the mucosal epithelium had movable and dense cilia, so the mucosal epithelium was a pseudostratified columnar ciliated epithelium. The mucosal epithelial cells were well-developed, consisting of columnar ciliated cells and a large number of secretory cells. A large number of glands were present in the lamina propria of the mucosa. Sparse and scattered collagen fibers were observed in the lamina propria. Collagen fibers are also embedded in smooth muscle (Figure 1A). However, the number of collagen fibers in the uterine-vaginal junction is less than in other parts of the chicken oviduct. The blood vessels contained a large number of red blood cells and were surrounded by a large number of collagen fibers (Figures 1C,D). The sperm storage tube existed between the collagen fibers, most of which were in the lamina propria, close to the mucosal epithelium (Figure 1A, black triangular arrow; Figure 1B, wavy arrow). The sperm storage tube was mostly round or oval. They were monolayer tubular glands surrounded by conical cells or monolayer columnar epithelium. The glands opened in the mucosal epithelium and were mainly distributed at the edge of the mucosal fold. Several glandular cells were close by but arranged irregularly. The gland was generally composed of 15–20 glandular cells.

### FISH Analysis of Telocytes in Chicken Utero-Vaginal Junction

**Figure 2:**
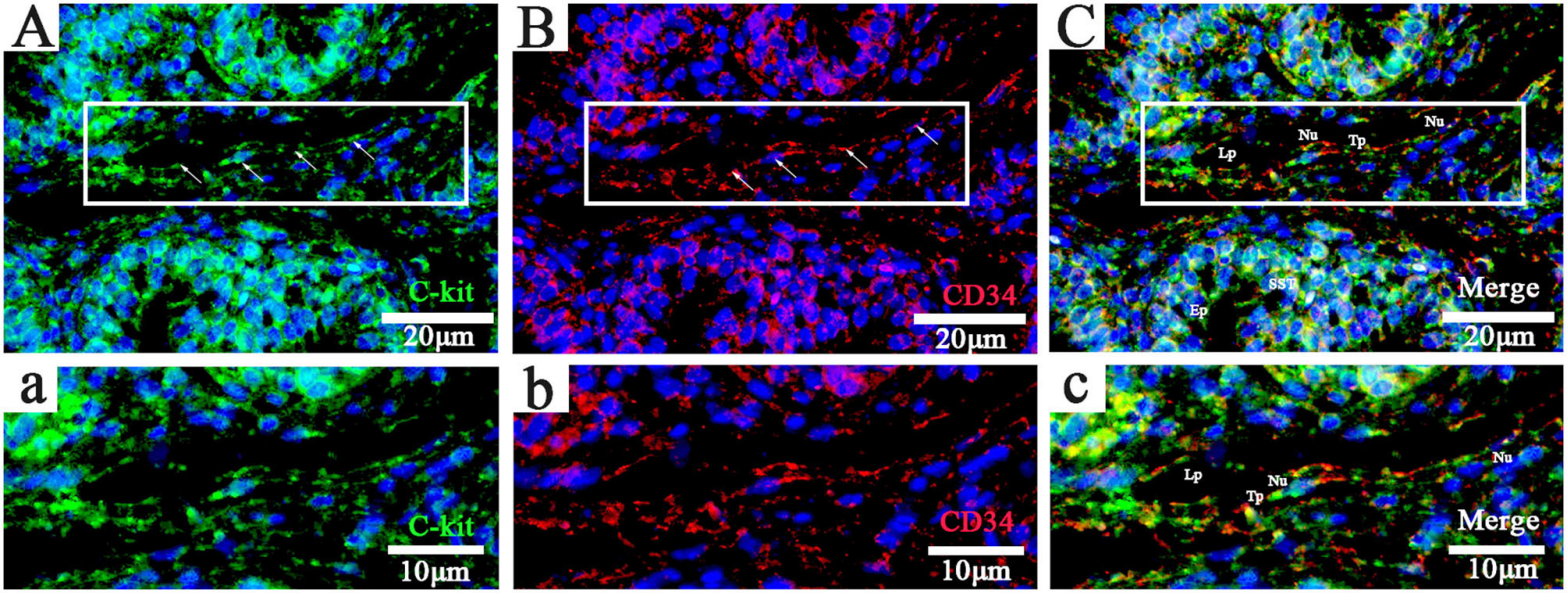
Fluorescence probe CD34/C-kit FISH in the uterine-vaginal junction of the oviduct in chicken. (**A–C**, a-c; Panels a, b, and c are enlarged versions of Panels A, B, and C, respectively: C-kit^+^, CD34^+^, and merged). DAPI (blue), C-kit^+^(green),CD34^+^(red). Lp, lamina propria; Nu, nuclear; SST, sperm storage tube; Tp, telopode (arrows); Ep, epithelial cells. Scale bar **(A–C)** = 20 μm; Scale bar (a-c) = 10 μm.

Chickens' utero-vaginal junction TCs were CD34/C-kit positive (Figure 2). The CD34/C-kit-positive reaction was mainly expressed in the lamina propria of the mucosal layer at the junction of the utero-vaginal junction and vagina. CD34/C-kit-positive cells were present around the subepithelial sperm storage tube and were attached to the peripheral glands – prolongations with dichotomous branching that bears spindle-shaped or cone-shaped cells with nucleus (Figures 2A–C, a–c). Due to the lack of commercial poultry antibodies on the market, we further identified TCs at the utero-vaginal junction using the “gold standard” (TEM) for identifying TCs.

### Ultrastructure of Telocytes in the Utero-Vaginal Junction of Chicken

**Figure 3:**
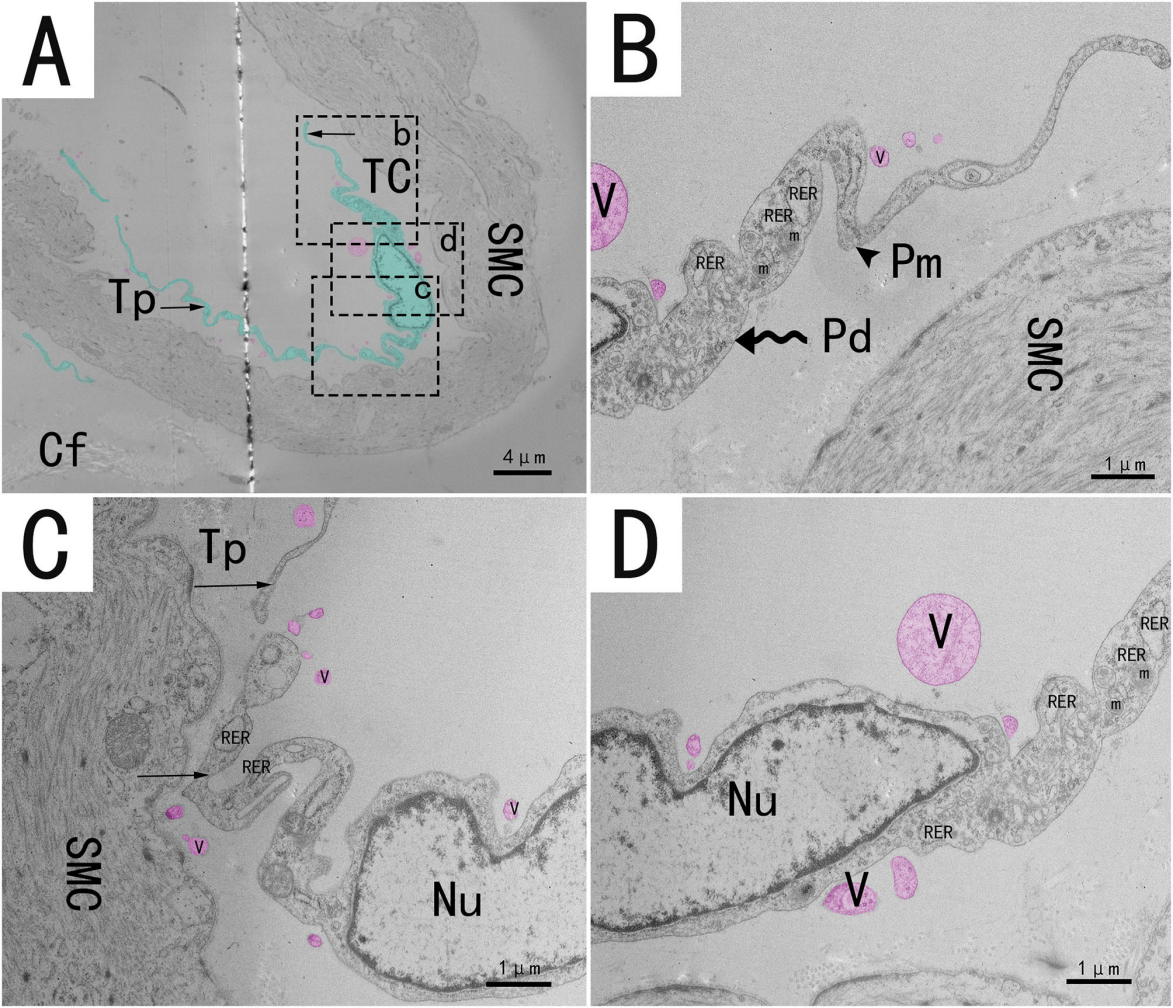
Transmission electron microscope analysis of TCs from the utero-vaginal junction of the chicken **(A)**. **(B–D)** The enlargement of the area marked by the dotted box in panels b–d. Around the smooth muscle, a typical TC has an irregularly fusiform nucleus and two long, curved and folded rosary projections containing alternating bulges (**B**, wavy arrow, Podom) and fine segments (**B**, black triangular arrow, Podomers). Sparsely distributed vesicles of different sizes and shapes can be seen around the protrusion (red mark in this figure). Mitochondria and rough endoplasmic reticulum were abundant in the enlarged region **(B–D)**. TC, telocyte; Tp, telopode (black arrow); Pd, Podom; Pm, Podomer; SMC, Smooth muscle; Cf, Collagen fiber; RER, rough endoplasmic reticulum; m, Mitochondria; V, vesicle. Scale bar **(A)** = 4 μm; Scale bar **(B–D)** = 1 μm.

**Figure 4:**
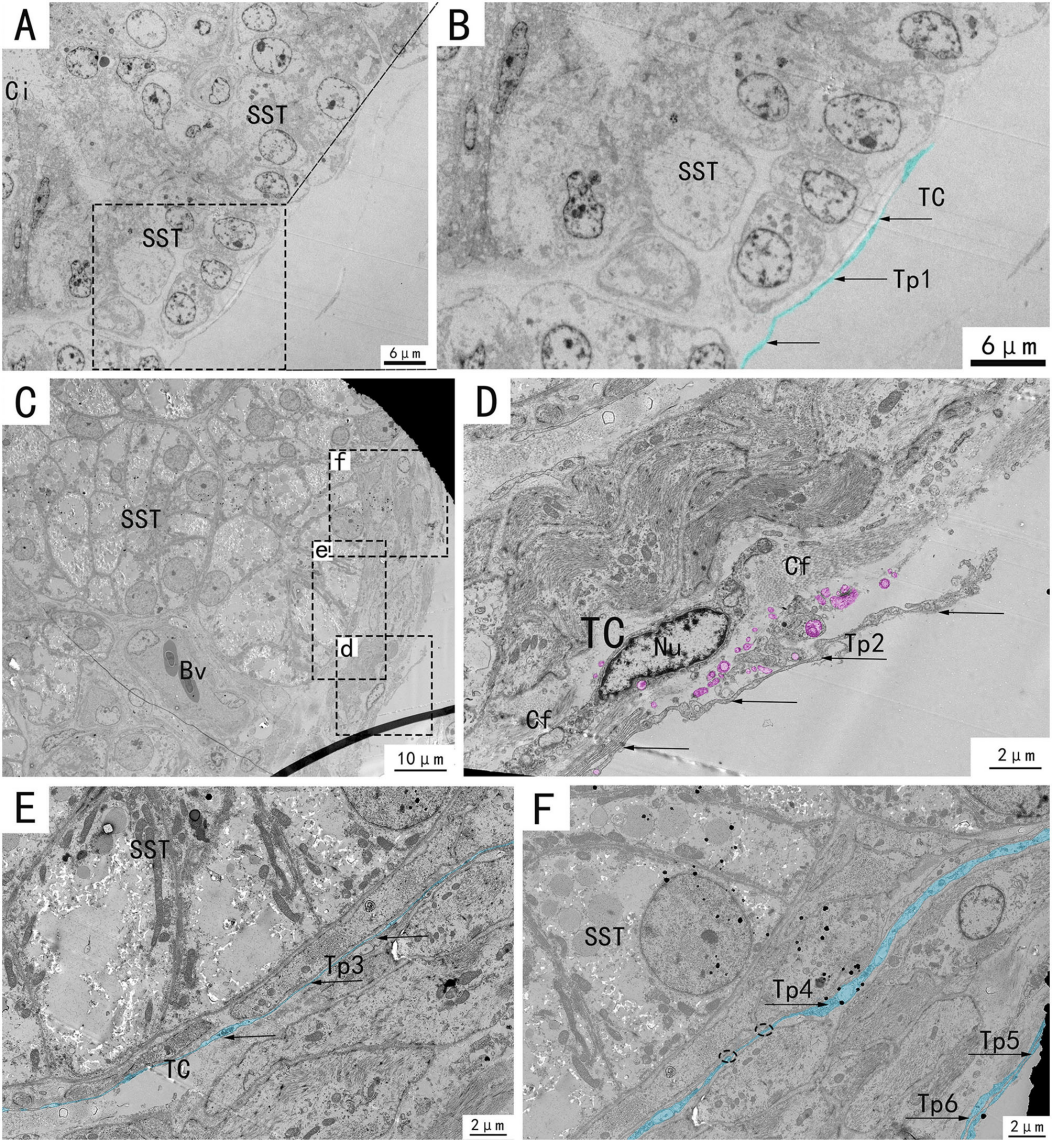
Transmission electron microscope analysis of glands and TCs in the utero-vaginal junction of the chicken. **(B,D–F)** The amplification of the area marked by the dotted box in **(A)**, d–f, respectively. TCs, blood vessels, and collagen fibers are present around the subepithelial glands **(C)**. A typical TC is located around the gland, embedded in collagen fibers, with a long fusiform nucleus and a long labyrinth of Tp surrounded by clearly visible vesicles of varying sizes, as well as synthetic but unreleased vesicles in Tp (**D**, marked pink). Two very elongated, almost parallel Tps surround the glands closely (**E,F**, marked in blue, with black arrows). SST, sperm storage tube; V, vesicle; Bv, blood vessel; telopode, Tp1, Tp2, Tp3, Tp4, Tp5, Tp6; Cf, Collagen fiber. Scale bar **(A,B)** = 6 μm; Scale bar **(C)** = 10 μm; Scale bar **(D–F)** = 2 μm.

**Figure 5:**
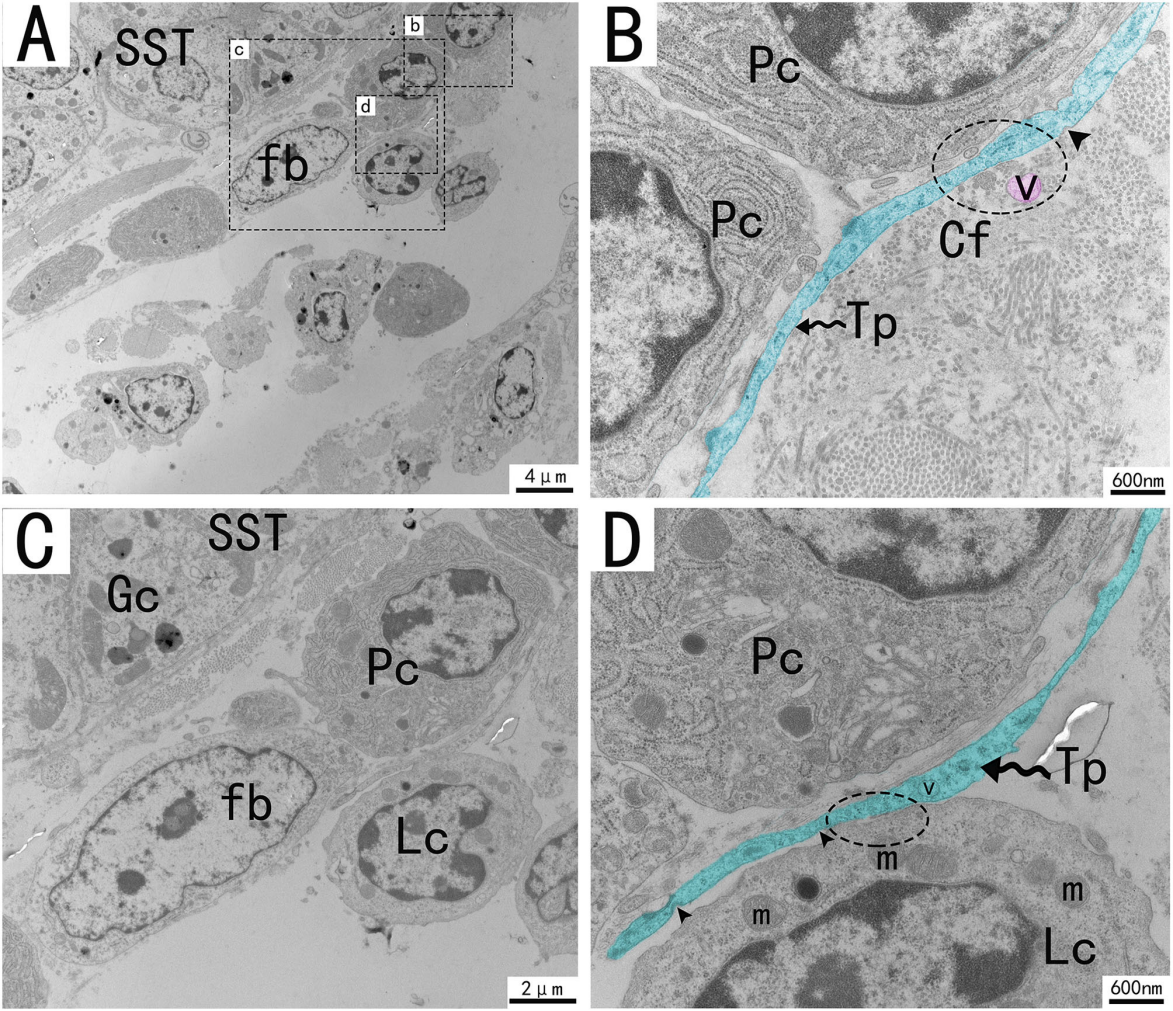
Transmission electron microscope analysis of glands, TCs, and adjacent cells in the utero-vaginal junction of the chicken **(A)**. **(B–D)** The enlargement of the area marked by the dotted box in panels b–d. A typical Tp of TC is surrounded by plasma cells, fibroblast lymphocytes, and collagen fibers. TCs form typical cell connections with surrounding plasma cells and lymphocytes through Tps, and there are a large number of mitochondria in the Podom (**B,D**, dotted ellipse). Around Tps, vesicles of uniform size and different shapes can be clearly seen and are scattered (**B**, marked in red). There were a large number of mitochondria and rough endoplasmic reticulum in the enlargement of Tps, and the unreleased vesicles were also clearly visible **(B,D)**. Several concave pits around the bulge are associated with calcium absorption and release (**D**, black triangular arrow). The gland is surrounded by plasma cells, fibroblasts, etc. The nucleus of the glandular cell is located in the center. Tp, telopodes (black wavy arrow); Cf, Collagen fiber; RER, rough endoplasmic reticulum; m, Mitochondria; Lc, lymphocytes; Pc, plasma cell; Fb, fibroblast; Gc, Gland cell; V, vesicles; SST, sperm storage tube. Scale bar **(A)** = 4 μm; Scale bar **(B,D)** = 600 nm; Scale bar **(C)** = 2 μm.

**Figure 6:**
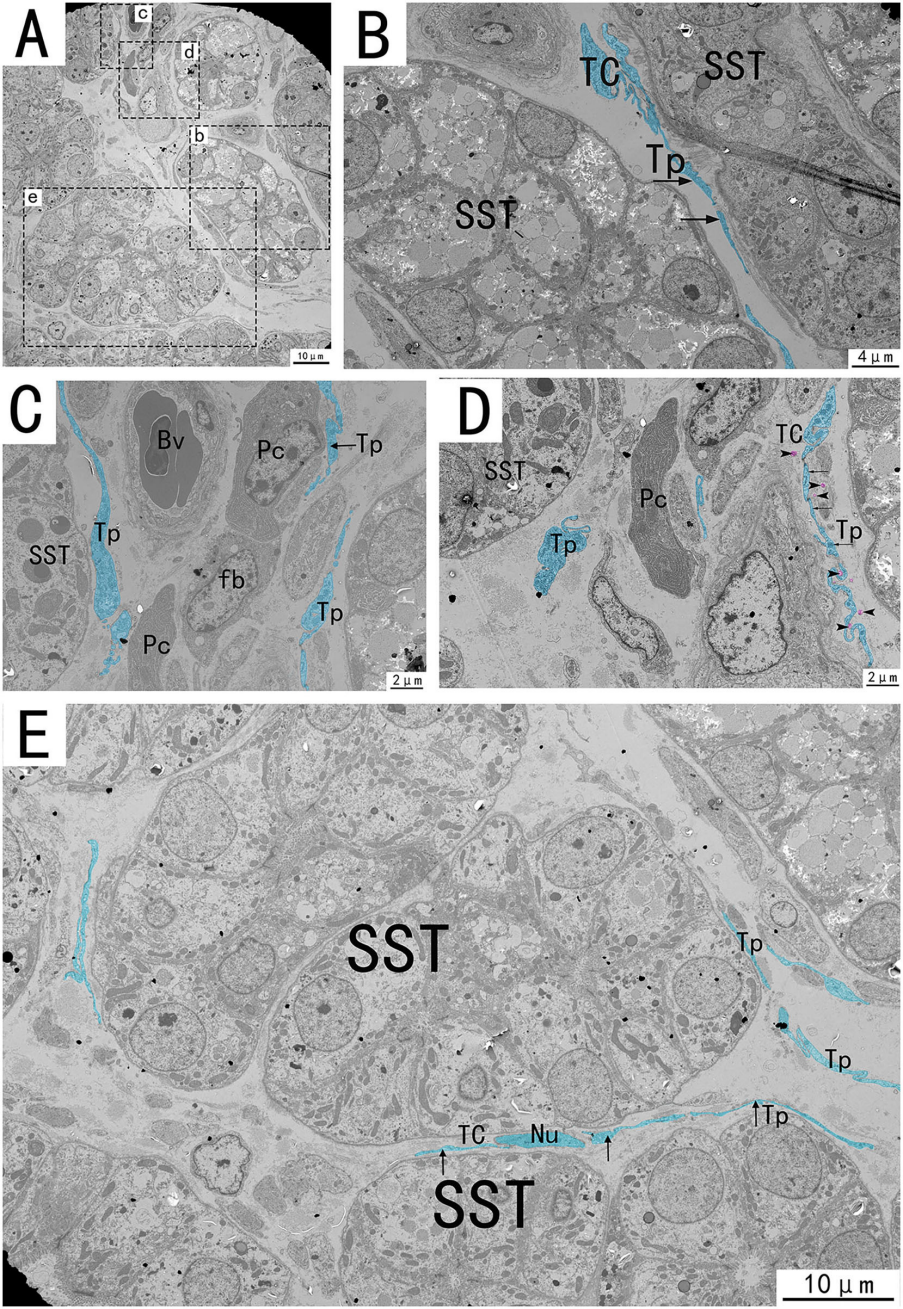
Transmission electron microscope analysis of glands, TCs, vessels, and adjacent cells in the utero-vaginal junction of the chicken **(A)**. **(B–E)** The amplification of the area marked by the dotted box in panels b–e. The typical TCs can be seen between the glands. Tp is curved and folded like a labyrinth, with long fusiform nuclei and long Tp surrounded by vesicles of varying sizes (**B,E**, black arrows). Tp is surrounded by collagen fibers. There is TC between the glands and the blood vessels, forming a SST-TC-Bv positional relationship **(C)**. There is TC between the gland and fibroblasts, forming a SST-TC-fb positional relationship **(D)**. There are also vesicles in the process of synthesis in Tp that have not yet been released (**D**, marked in pink). SST, sperm storage tube; Bv, blood vessel; Cf, Collagen fiber; Pc, plasma cell; Fb, fibroblast; Nu, nuclear. Scale bar **(A,E)** = 10 μm; Scale bar **(B)** = 4 μm; Scale bar **(C,D)** = 2 μm.

**Figure 7:**
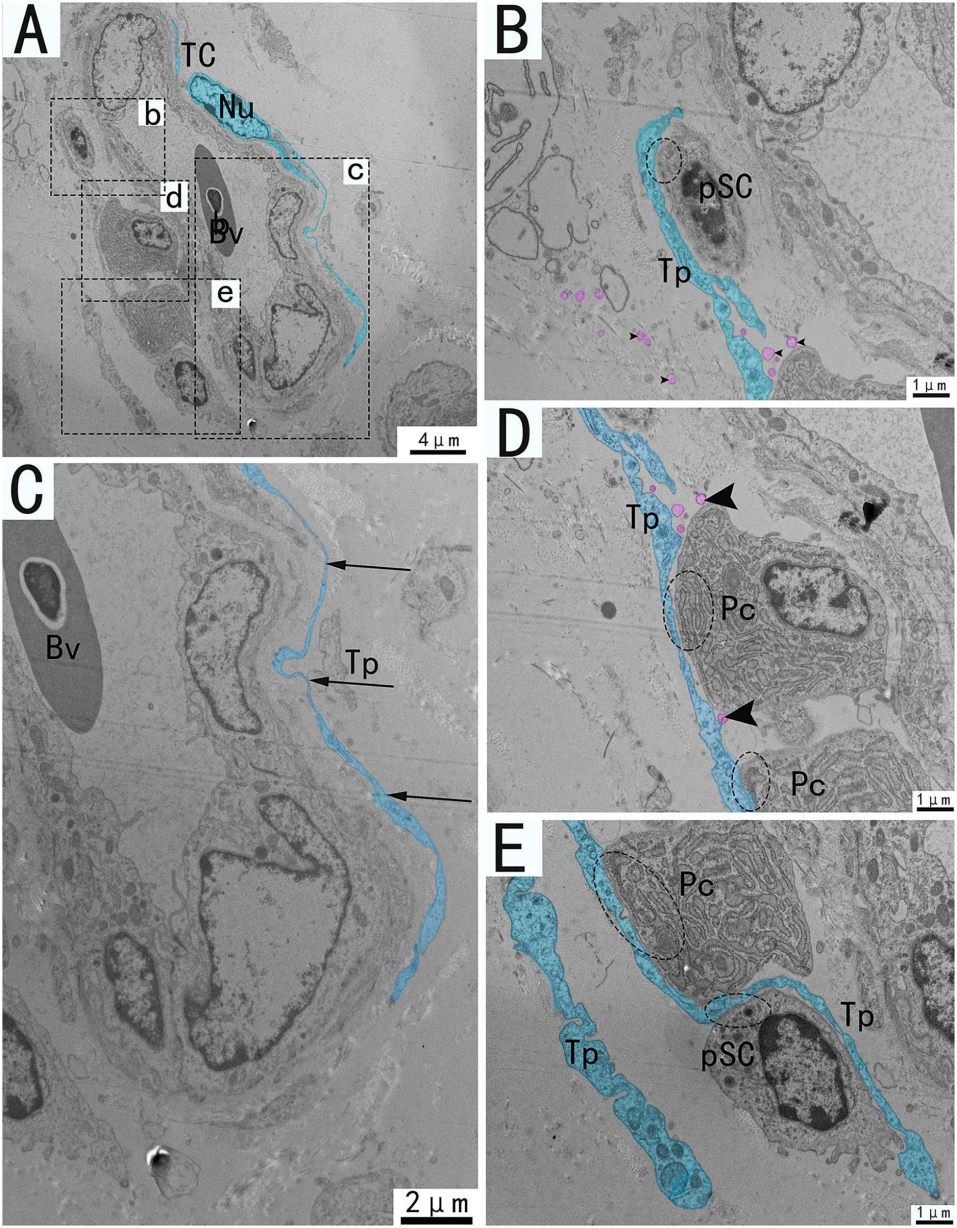
Transmission electron microscope analysis of TCs, vessels, and adjacent cells from the utero-vaginal junction of the chicken **(A)**. **(B–E)** The enlargement of the area of the dotted box in panels b–e. Around the blood vessels, TCs forms homotypic cell connections *via* Tp (**A**, dashed ellipse). The vessels are typically surrounded by TCs and Tp is extremely elongated **(C)**. **(B,D,E)** Tp of the same TC forms heterogeneous cell connections with plasma cells, fibroblasts, stem cells, etc. (**B,D,E**, dotted ellipse), forming a network structure connecting blood vessels and surrounding cells in the stroma. There are vesicles of different sizes scattered around Tp (**B,D**, black triangular arrow), and there are also vesicles in the process of synthesis that have not been released (**D**, pink marks). Bv, blood vessel; Pc, plasma cell; pSC, potential stem cells; Tp, telopode; Nu, nuclear. Scale bar **(A)** = 4 μm; Scale bar **(C)** = 2 μm; Scale bar **(B,D,E)** = 1 μm.

Transmission electron microscopy (TEM) showed that TCs were present in both the muscularis and lamina propria of the utero-vaginal junction and had a typical TCs cell structure (Figures 3–7). The muscularis layer of the utero-vaginal junction was well developed, and TCs were observed in the interstitial spaces between the smooth muscle bundles (Figure 3). The Tps were slender and rosary shaped with alternating enlarged and elongated segments (Figure 3A). The enlarged segments contained abundant mitochondria, rough endoplasmic reticulum, and vesicles, whereas few organelles were present in the elongated segments (Figures 3B–D). The TCs cell body is small and spindle-shaped, with a spindle or oval nucleus with protrusions extending tens or even hundreds of microns long (Figures 3–7). TCs are located around the gland (Figures 4A,B, 6A,B,E), embedded in collagen fibers (Figures 4E,F), with a long fusiform nucleus and a long labyrinth of Tp surrounded by clearly visible vesicles of varying sizes (Figures 4C,D), as well as synthetic but unreleased vesicles in Tps. The elongated protrusions allowed the TCs to contact surrounding TCs, sperm storage tube, smooth muscle cells, blood vessels, plasma cells, potential stem cells, and lymphocytes to form homomorphic or heteromorphic connections and a complex 3D network structure, which play a role in information communication and transmission (Figures 4–7). TCs form a typical cell connection with surrounding plasma and lymphocyte cells through Tps, and there are a large number of mitochondria in the Podom (Figures 5B,D). Around the blood vessels, TCs form homotypic cell connections *via* Tps (Figures 7B–E).

## Discussion

Since TCs were discovered by Professor Popescu in 2005, they have attracted extensive attention from many researchers. The markers expressed in TCs vary from tissue to tissue, and even from cell to cell within the same tissue (2). It is difficult to observe the typical morphological characteristics of TCs under light microscope. So, TEM is the “gold standard” for identifying TCs and the only accurate method to identify TCs at present (24). Connections between TCs and homotypic or heterotypic cells can also be observed using TEM (25).

In this study, a combination of TEM and FISH (CD34 and C-kit) was used (16, 26) to reveal the existence of TCs at the utero-vaginal junction of the chicken for the first time. TCs at the utero-vaginal junction are consistent with previous studies. Chicken utero-vaginal junction TCs exhibited CD34 and C-kit immunopositive by FISH. TCs express CD34, which is frequently expressed in hematopoietic stem/progenitor cells. It is suggested that TC has the dryness and properties of undifferentiated cells. Morphologically, the TCs in the chicken utero-vaginal junction have a slender Tp and can also secrete vesicles, which is consistent with the structure of typical TCs.

Our TEM results show two or three slender Tps were observed on TEM. The number of Tps depended on the angle and position of the section, as all Tps cannot be completely present on a plane during sectioning (27, 28). These structures can only be observed by TEM, such as the identification criteria proposed by Professors Popescu and Faussone-Pellegrini (29). Masson's staining showed that the utero-vaginal junction was composed of glandular cells, epithelial cells, secretory cells, ciliary cells, collagen fiber bundles, smooth muscle cells, immunoreactive cells, and blood vessels. The secretory and ciliated cells produce tubular fluid and promote gamete transport, respectively (30). Masson's staining showed that the lamina propria of the utero-vaginal junction was thinner and had few collagen fibers. The number of TCs was positively correlated with the number of lamina propria cells, and there were fewer TCs in this part compared with other animals or tissues and organs. There are still some differences between avian and mammalian oviduct TCs. TCs were mainly distributed in the mucosa and muscular layer between smooth muscle fibers in the human oviduct (14), while TCs in the utero-vaginal junction of the chicken existed in the area below the epithelium and lamina propria, between collagen fibers and smooth muscle bundles, and around blood vessels and some glands.

The unique morphology and structure of TCs must allow for unique function. TCs may play a role in signaling between cells. The stroma is composed of cells that integrate all information from the blood vessels, nerves, the immune system, and stem cells through contact with the same or different cells (20). TCs use their elongated protrusions to form homotypic or heterotypic connections with adjacent cells (31) and produce a 3D network structure in the stroma. The blood vessels and glands in the utero-vaginal junction were surrounded by TCs, and also occurred between collagen fiber bundles and smooth muscle. The TCs formed homotypic and heterotypic connections with other TCs, potential stem cells, plasma cells, fibroblasts, and glandular cells. It has been proposed that TCs function in cell-to-cell communication by paracrine secretion of small molecules, or by the release of cellular vesicles that transport important macromolecules (3, 32–34). Cellular vesicles secreted by telocytes contain mainly proteins, lipids, microRNAs, mRNAs, and mitochondrial DNA (mtDNA), indicating a key role of these cells in intercellular signaling of the interstitial compartment, influencing the function and/or modification of post-transcriptional activity of neighboring cells (35, 36). In the heart, TCs release three types of extracellular vesicles, such as exosomes, microvesicles, and polyvesicles (31), but the exact functions of the vesicles after release remain to be determined. Scattered vesicles were detected around the TCs at the utero-vaginal junction, and vesicles used in the process of synthesis and secretion by TCs were also present, providing morphological support for information communication between cells. These results suggest that the vesicles and exosomes released by TCs at the utero-vaginal junction may be involved in intercellular communication.

TCs were located around capillaries and connected with fibroblasts and pericytes. TCs also express growth factor receptors involved in neovascularization (37–39). TCs that make heterocytic contacts with various stromal components in the oviduct may participate in information exchange between various stromal cells or may participate in information exchange through the indirect structure of the TCs-vesicle gap junction-cytoskeleton (40, 41). TCs that function in specific intercellular signaling help regulate the activity of adjacent cells and regulate tissue development, remodeling, metabolism, immune regulation, immune monitoring, and maintenance of gastrointestinal homeostasis (42, 43).

The ICCs in the gastrointestinal tract have the role of slow-wave pacing and conduction (44). TCs form an intercellular 3D network structure in the intestinal muscular layer that resists deformation and thus supports gastrointestinal peristalsis (45). Similarly, the network of TCs creating a 3D scaffold in the human urinary bladder interstitial space provides mechanical support during bladder wall expansion and relaxation and avoids abnormal wall deformation (46). Tubal TCs also produce slow waves in the smooth muscle of the oviduct and are considered pacemaker cells of the oviduct (40, 41, 47). TCs express estrogen and progesterone receptors in the human myometrium and act as estrogen sensors to participate in myometrial contraction and movement of substances in the oviduct through gap junction or paracrine mechanisms (47, 48). These results provide morphological evidence for the role of TCs in the contractive motion of smooth muscle at the uterine-vaginal junction and in promoting the transport of sperm and ovum.

TCs have also been found in the intestinal crypt of the exocrine pancreas and intestinal stem cells (49, 50). TCs may be a subpopulation of mesenchymal progenitor/dry cells and thus have the potential to differentiate into other cell types. The formation of heterocellular connections between TCs and potential stem cells around the gland suggests that TCs are involved in epithelial renewal and play an important role in the repair and regeneration of the utero-vaginal junction. Cretoiu and Popescu reported that TCs in the human mammary gland and myometrium, and in the rat stomach, intestine, bladder, and uterus, establish close contacts with various immune cells, including lymphocytes, plasma cells, eosinophils, basophils, macrophages, and mast cells (2, 51–53). A large number of glands and sperm storage glands exist, and TCs surround these glands, forming heteromorphic cell connections with surrounding lymphocytes and plasma cells, and have an immune function in the microenvironment of the utero-vaginal junction.

In conclusion, our results confirm the presence of TCs at the utero-vaginal junction in chickens. TCs were CD34/C-kit positive. TCs surrounds glands, forming homogenous or heterogenous connections with lymphocytes, plasma cells, smooth muscle cells, blood vessels, collagen fibers, and fibroblasts around them, forming a complex three-dimensional network structure. This study provides morphological basis for the possible role of TCs in regulating utero-vaginal junction physiological function and intercellular communication.

## Conclusions

This is the first study to confirm the existence of TCs at the utero-vaginal junction of the chicken. In the lamina propria, the TCs were curved and folded, embedded between bundles of collagen fibers and around blood vessels, and released vesicles. In the smooth muscle layer, TCs were mostly distributed around smooth muscle bundles. TCs and their surrounding structures, such as sperm storage tube, smooth muscle cells, blood vessels, collagen fibers, plasma cells, and fibroblasts, formed homotypic and allotypic connections to produce a 3D network structure. We postulate that TCs may play a role in information transmission, which provides a theoretical basis for the relationship between TCs in the utero-vaginal junction and sperm storage, transport, and oviduct disease, and provides a reference for the plasticity of TCs. This study provides morphological basis for the possible role of TCs in regulating the utero-vaginal junction physiological function and intercellular communication.

## Data Availability Statement

The original contributions presented in the study are included in the article/supplementary material, further inquiries can be directed to the corresponding author/s.

## Ethics Statement

The animal study was reviewed and approved by the Science and Technology Agency of Jiangsu Province. Written informed consent was obtained from the owners for the participation of their animals in this study.

## Author Contributions

XZ, QW, PP, and PY: conceptualization, data curation, and writing—review and editing. ZW and BP: formal analysis. ZW, BP, XM, YF, and PY: methodology. XZ, QW, and PP: writing—original draft. All authors have read and agreed to the published version of the manuscript.

## Funding

This work was supported by the National Natural Science Foundation of China (Grant No. 31772688), and the Priority Academic Program Development of Jiangsu Higher Education Institutions, China.

## Conflict of Interest

The authors declare that the research was conducted in the absence of any commercial or financial relationships that could be construed as a potential conflict of interest.

## Publisher's Note

All claims expressed in this article are solely those of the authors and do not necessarily represent those of their affiliated organizations, or those of the publisher, the editors and the reviewers. Any product that may be evaluated in this article, or claim that may be made by its manufacturer, is not guaranteed or endorsed by the publisher.
